# Factors associated with modern contraceptive utilization among reproductive age women in Kenya; evidenced by the 2022 Kenyan demographic and health survey

**DOI:** 10.1186/s40834-024-00271-1

**Published:** 2024-03-15

**Authors:** Gosa Mankelkl, Altaseb Beyene Kassaw, Beletu Kinfe

**Affiliations:** 1 Department of Biomedical Sciences, College of Medicine and Health Science, Wollo University, Dessie, Ethiopia; 2 Department of occupational Health and safety, College of Medicine and Health Science, Wollo University, Dessie, Ethiopia

**Keywords:** Prevalence, Modern contraceptive, Kenya

## Abstract

**Background:**

Globally, sexual and reproductive health is a significant public health issue for women of the reproductive age group. A modern contraceptive method enables individuals and families to manage fertility by reducing unintended pregnancies, abortions, pregnancy-related morbidity, and death. A modern contraceptive method is a drug or medical treatment that prevents sexual activity from leading to pregnancy. However, there is limited reliable and updated data on factors associated with modern contraceptive utilization among reproductive-age women at the national level in Kenya. So, the major goal of this study was to evaluate factors associated with modern contraceptive utilization among women of reproductive age in Kenya at the national level, as evidenced by the 2022 Kenyan demographic and health survey.

**Methods:**

The most recent datasets from the Kenyan Demographic and Health Survey were used for secondary data analysis. In all, 14,987 women of reproductive age participated in the investigation. Data for multivariable analysis on the factors influencing modern contraceptive utilization among Kenyan women of reproductive age can be obtained from the Kenyan Demographic and Health Survey. Finally, the odd ratio and percentages were presented along with their 95% confidence intervals.

**Result:**

This study includes a total weighted sample of 14,987 reproductive-age women from the Kenyan demographic and health survey. Of the total contraceptive use, 90.1% of the study participants used modern contraceptives. Being married [AOR: 1.593, 95% CI (1.302, 1.948)], living in an urban area [AOR: 1.230, 95% CI (1.060, 1.428)], reading a magazine [1.002, 95% CI (0.921, 1.091)], listening to radio [AOR: 1.265, 95% CI (1.101, 1.454)], not breastfeeding [AOR: 1.296, 95% CI (1.114, 1.507), and having more than two children [AOR: 2.350, 95% CI (1.603, 3.445)] were the factors that promote modern contraceptive utilization. Conversely, having a history of terminated pregnancy [AOR: 0.767, 95% CI (0.657, 0.897), being Muslim [AOR: 0.566, 95% CI (0.418, 0.766)], and being in the 35–39 age range [AOR: 0.766, 95% CI (0.605, 0.971)] were all associated with a lower use of modern contraceptives.

**Conclusion:**

Certain factors such as marriage, living in urban areas, having more than two children, having a female-led household, belonging to the middle class, reading magazines, listening to the radio, and not breastfeeding have a positive correlation with the use of modern contraceptives. Conversely, being a Muslim, aged between 35 and 39, and having a history of miscarriages are negatively correlated with the use of modern contraceptives. This indicates that addressing socioeconomic, geographic, and cultural barriers could improve the effectiveness of modern contraceptive.

## Introduction

Contraception enables individuals and families to manage fertility by reducing unintended pregnancy, abortions, pregnancy-related morbidity, and death [1]. Contraceptive method is a practice that helps individuals or couples to attain their desired children as their plan, and available methods of contraception should be customized to individual needs with a range of options that are acceptable to all [2]. A modern contraceptive method is a drug or medical treatment that prevents sexual activity from leading to pregnancy. Modern contraceptive methods include barrier methods such as male and female condoms, diaphragms, cervical caps, and sponges; hormonal contraceptives that include oral, injectable, transdermal, vaginal ring, and implants; and intrauterine devices (IUD) [3, 4]. Hormonal methods of birth control (contraception) contain either estrogen and progestin or progestin-only; they are a safe and reliable way to prevent pregnancy for most people [5].

Globally, 1.9 billion women between the ages of 15 and 49 require family planning, which is predicted to rise. Of them, 842 million utilize contraceptives, and 270 million still need contraceptives [6]. A modern contraceptive method of family planning was used by 58% of married or in-union women of reproductive age worldwide in 2017, accounting for 92% of all contraceptive users [7]. Substantial progress has been made in Kenya toward achieving universal access to FP. But women’s unmet need for FP continues [8, 9]. Pregnancies in Kenya are unplanned in 43% of cases. Adolescents have the greatest unmet need for family planning [10]. Globally, sexual and reproductive health is a significant public health issue for women of reproductive age. Women in low and middle-income countries (LMICs) are at higher risk of dying due to pregnancy-related complications [11, 12]. Numerous studies have demonstrated that the utilization of modern contraceptives has a significant association with factors such as marital status, number of living children, religion, education, and place of residence [13–15].

Several studies were conducted in Kenya related to modern contraceptive utilization in different settings with small sample sizes. However, few studies were conducted at the national level. In addition to this, the Kenyan DHS reports simply report the proportion or frequency of certain events without considering the factors associated with them. The major objective of this study was to evaluate factors associated with modern contraceptive utilization among reproductive women in Kenya at the national level by using the Kenyan demographic and health survey. The findings of this study would also provide better evidence for policymakers and other stakeholders, which in turn might enable the designing and execution of appropriate intervention programs at different levels to promote modern contraceptive utilization that reduces maternal and child morbidity and mortality.

## Methods and material

### Study setting and period

Kenya is a country located in East Africa with a population of over 47.6 million, according to the 2019 census. It is the 28th most populous country in the world and the 7th most populous in Africa. Nairobi is Kenya’s capital and largest city. Kenya shares borders with South Sudan to the northwest, Ethiopia to the north, Somalia to the east, Uganda to the west, Tanzania to the south, and the Indian Ocean to the southeast [16]. The 2022 Kenya Demographic and Health Survey (2022 KDHS) was implemented by the Kenya National Bureau of Statistics (KNBS) in collaboration with the Ministry of Health (MoH) and other stakeholders. This is the 7th KDHS implemented in the country. Data collection took place from 17 February to 31 July 2022 [17].

### Data source/extraction

After permission was secured through an online request explaining the aim of the study, the data were taken from the Measure Demographic and Health Surveys (DHS) website (http://www.dhsprogram.com/).

### Study design

A community-based cross-sectional study design was employed. The 2022 KDHS employed a two-stage stratified sample design, where in the first stage 1,692 clusters were selected from the Kenya Household Master Sample Frame (K-HMSF) using the Equal Probability Selection Method (EPSEM). The clusters were selected independently in each sampling stratum. Household listing was carried out in all the selected clusters, and the resulting list of households served as a sampling frame for the second stage of selection, where 25 households were selected from each cluster. Therefore, all households from these clusters were selected for the sample. This resulted in 42,022 households being sampled for the 2022 KDHS. Details about the study design and sampling techniques were available in the final Kenyan demographic health survey reports [17].

### Study population

All women aged 15–49 who were either permanent residents of the selected households or visitors who stayed in the household the night before the survey were eligible to be interviewed. The Woman’s Questionnaire was used to collect information from all eligible women aged 15–49. The total number of eligible women that were interviewed was 32,156. 13,143 of the 32,156 women were from an urban area, as opposed to 19,013, who were from a rural area. Of the total interviewed women, 14,987 used contraceptives, and the remaining 17,169 didn’t. So, they were excluded from this analysis. Modern contraceptive utilization among women of reproductive age was the outcome variable for this study. So, the total number of samples used in this analysis was 14,987 [17].

### Study variables

**The outcome variable** for this study was modern contraceptive utilization. If the women utilized modern contraceptives, which were coded as “0,” if they utilized another method of contraceptive (traditional and folkloric), it was coded as “1.”.

#### Predictor variables

Age group, place of residence, educational status, marital status, religion, sex of household head, wealth index, reading newspapers, listening to radio, watching television, history of terminated pregnancy, currently breastfeeding, and number of children.

### Data management and analysis

In all of our analyses, we took weighting, stratification, and clustering into account to consider the complex survey design. We pooled and compared data from different regions with varying target population sizes to normalize the weights. We divided the national total of women and the standard weights of women by the proportion of survey samples that corresponded to them to arrive at this result. We used STATA version 14 for data extraction, recording, and both descriptive and analytical analysis. We conducted bivariate analyses on various variables, including age group, place of residence, educational status, marital status, religion, sex of household head, wealth index, reading newspapers, listening to radio, watching television, history of a terminated pregnancy, current breast-feeding, and number of children. We used the results of the bivariate analysis to select variables for the multivariate analysis. For the multivariate analysis, we only considered factors with *p*-values of less than 0.05.

### Ethical consideration

Since the secondary survey data we used for this investigation came from readily available demographic and health survey programs, neither an ethical review nor participant agreement were required. We received authorization from the DHS Program to utilize the information we collected from their website, and they provided us with access to their website.

### Socio-demographic factors of the study participants

A total of 14,987 reproductive age women were involved in this study. About 3451 (23.0%) were found between the age ranges of 15–19 years old. The majority of respondents 8740 (58.3%) were live in rural area, 6208(41.4%) were protestant, 6004(40.1%) were attending primary educations, 9567(63.8%) were married, 10,180(67.9%) were male headed households, 10,967(73.2%) were poor, 3014(20.1%) were reading magazine, 11,360 (81.2%) were listening radio, 10,422(69.5%) were watching television, 2099 (14.0%) had history of terminated pregnancy, 3698 (24.7%) were breast feeding, had less than or equal to two children14347(95.7%) and from the total contraceptive utilizer, modern contraceptive utilization accounts for 13,501 (90.1%) (Table 1).

**Table 1:** Socio demographic characteristics of reproductive age women in Kenya 2023

Characteristics	Categories	Modern contraceptive		Frequency	Percentage
		Yes	No		
Age group	15–19	3016	435	3451	23.0
	20–24	3141	265	3406	22.7
	25–29	2531	222	2753	18.4
	30–34	2342	230	2571	17.2
	35–39	1533	206	1739	11.6
	40–44	938	129	1066	7.1
Place of residence	Urban	5504	744	6247	41.7
	Rural	7998	742	8740	58.3
Religion	Catholic	3306	315	3621	24.2
	Protestant	5557	651	6208	41.4
	Evangelical churches	3087	283	3370	22.5
	African instituted churches	954	138	1092	7.3
	Islam	169	8	177	1.2
	Other	428	91	519	3.5
Educational status	No education	347	65	347	2.7
	Primary	5613	391	5613	40.1
	Secondary	4787	488	4787	35.2
	Higher	2755	542	2755	22.0
Marital status	Never in union	1867	356	2223	14.8
	Married	8719	848	9567	63.8
	Widowed	253	20	273	1.8
	Divorced	135	26	161	1.1
	Other	2527	237	2764	18.4
Sex of house hold head	Male	9243	937	10,180	67.9
	Female	4258	549	4807	32.1
Wealth index	Poor	10,001	966	10,967	73.2
	Middle	3500	520	4020	26.8
Frequency of reading newspaper	No	10,884	1089	11,973	79.9
	Yes	2617	397	3014	20.1
Frequency of listening radio	No	2488	326	2814	18.8
	Yes	11,013	1160	12,173	81.2
Frequency of watching television	No	4166	400	4566	30.5
	Yes	9335	1086	10,421	69.5
History of terminated pregnancy	No	11,638	1250	12,888	86.0
	Yes	1863	236	2099	14.0
Currently breast feeding	No	10,074	1215	11,289	75.3
	Yes	3427	271	3698	24.7
Number of children	=<2	12,890	1457	14,347	95.7
	> 2	611	29	640	4.3
Contraceptive utilization	Modern method	13,501	0	13,501	90.1
	Other method	0	1486	1486	9.9

### Factors analysis associated with modern contraceptive utilization

Age group, place of residence, educational status, marital status, religion, sex of household head, wealth index, reading newspapers, listening to radio, watching television, history of a terminated pregnancy, current breastfeeding, and number of children were all taken into account in the bivariate analysis. The results of the bivariate analysis showed that among women of reproductive age, modern contraceptive utilization was statistically and significantly associated with age group, place of residence, educational status, marital status, religion, sex of household head, wealth index, reading newspapers, listening to radio, watching television, history of a terminated pregnancy, currently breastfeeding, and number of children.The multivariable logistic regression analysis also revealed that age group, place of residence, educational status, marital status, religion, sex of household head, wealth index, reading newspapers, listening to radio, watching television, history of terminated pregnancy, currently breastfeeding, and number of children were significantly associated with modern contraceptive utilization among reproductive-age women.

The results of this study reveal that, when compared to women between the ages of 15 and 19, the odds of modern contraceptive utilization were 0.766 times lower among those between the ages of 35 and 39 [AOR: 0.766, 95% CI (0.605, 0.971); *P* = 0.010]. Women living in urban areas were 1.230 times more likely to use modern contraceptives than women living in rural areas [AOR: 1.230, 95% CI (1.060, 1.428); *P* = 0.006]. In comparison to women who were Catholic, the odds of modern contraceptive usage were 0.566 times lower among Islamic women [AOR: 0.566, 95% CI (0.418, 0.766); *P* = 0.000].

In comparison to male-heeded households, female-headed households had a 1.016-fold higher likelihood of using modern contraceptives [AOR: 1.016(0.892, 1.158); *P* = 0.042]. Among married women, the probability of using modern contraception was 1.593 times higher [AOR: 1.593, 95% CI (1.302, 1.948); *P* = 0.000] in comparison to women who had never been married. In comparison to women with a poor wealth index, the odds of modern contraceptive usage were 1.084 more likely among those with a middle wealth index [AOR: 1.084 (0.920, 1.277); *P* = 0.001].

Women who read magazines [AOR: =1.002 (0.921, 1.091); *P* = 0.015] and listed radio [AOR: 1.265 (1.101, 1.454); *P* = 0.004] had higher odds of using modern contraceptives than their counterparts, respectively. Women with a history of pregnancy termination were 0.767 times less likely to use modern contraceptives [AOR: 0.767 (0.657, 0.897); *P* = 0.007] than women without a history of pregnancy termination. Compared to women who were breastfeeding, the likelihood of modern contraceptive use among currently non-breastfeeding women was 1.296 times higher [AOR: 1.296, 95% CI (1.114, 1.507; *P* = 0.000)]. When compared to women who had less than or equal to two children, the likelihood of modern contraceptive usage was 2.350 more likely among those who had more than two children [AOR: 2.350, 95% CI (1.603, 3.445); *P* = 0.000] (Table 2).

**Table 2:** Bivariate and multivariate analysis of factors associated with modern contraceptive utilization among reproductive age women in Kenya 2023

Characteristics	Categories	Modern contraceptive utilization		COR with 95% CI and *P*-value	AOR with 95% CI and *P*-value
		Yes	No		
Age group	15–19	3016	435	1	1
	20–24	3141	265	1.049(0.851,1.294); 0.211	0.972(0.758,1.247);0.008
	25–29	2531	222	0.613(0.491,0.766); 0.000	0.601(0.472,0.765);0.000
	30–34	2342	230	0.640(0.508,0.805); 0.000	0.653(0.512,0.832);0.000
	35–39	1533	206	0.714(0.568,0.897);0.003	0.766(0.605,0.971);0.010
	40–44	938	129	0.978(0.773,01.237);0.687	1.025(0.804,1.305);0.848
Place of residence	Urban	5504	744	1.456(1.308,1.621);0.000	1.230(1.060,1.428);0.006
	Rural	7998	742	1	1
Religion	Catholic	3306	315	1	1
	Protestant	5557	651	0.450(0.349,0.581); 0.000	0.425(0.326,0.554);0.000
	Evangelical churches	3087	283	0.553(0.435,0.704);0.000	0.539(0.419,0.693);0.000
	African instituted churches	954	138	0.433(0.335,0.560); 0.000	0.416(0.318,0.544);0.000
	Islam	169	8	0.684(0.513,0.913);0.046	0.566(0.418,0.766);0.000
	Other	428	91	0.202(0.093,0.440); 0.001	0.207(0.094,0.455);0.000
Educational status	No education	347	65	1	1
	Primary	5613	391	0.959(0.725,1.268);0.635	1.164(0.835,1.620);0.832
	Secondary	4787	488	0.354(0.309,0.407);0.000	0.448(0.727,0.530);0.000
	Higher	2755	542	0.518 (0.454, 0.591);0.000	0.591(0.513,0.682);0.000
Marital status	Never in union	1867	356	1	1
	Married	8719	848	2.035(1.709,2.424);0.000	1.593(1.302,1.948);0.000
	Widowed	253	20	1.039(0.893,1.207);0.562	1.064(0.904,1.252);0.704
	Divorced	135	26	0.836(0.519,1.346);0.867	0.938(0.570,1.542);0.441
	Other	2527	237	2.045(1.315,3.181);0.707	2.208(1.394,3.497);0.900
Sex of house hold head	Male	9243	937	1	1
	Female	4258	549	0.787(0.704,0.880);0.000	1.016(0.892,1.158);0.042
Wealth index	Poor	10,001	966	1	1
	Middle	3500	520	0.650(0.580,0.728);0.004	1.084(0.920,1.277);0.001
Reading newspaper	No	10,884	1089	1	1
	Yes	2617	397	1.318(1.224,1.420);0.000	1.002(0.921,1.091);0.015
Listening radio	No	2488	326	1	1
	Yes	11,013	1160	1.242(1.090,1.415);0.000	1.265(1.101,1.454);0.004
Watching television	No	4166	400	1	1
	Yes	9335	1086	0.825(0.731,0.930);0.031	0.973(0.845,1.121);0.923
History of terminated pregnancy	No	11,638	1250	1	1
	Yes	1863	236	0.847(0.731, 0.981);0.013	0.767(0.657,0.897);0.007
Currently breast feeding	No	10,074	1215	1.528 (1.332, 1.753);0.000	1.296(1.114,1.507);0.000
	Yes	3427	271	1	1
Number of children	=<2	12,890	1457	1	1
	> 2	611	29	2.362 (1.623,3.436);0.000	2.350(1.603,3.445);0.000

COR = crudes odds ratio, AOR = adjusted odds ratio; CI-confidence interval; statistically significant at *P < 0.05; **P < 0.01; ***P < 0.001

## Discussions

The multivariable logistic regression analysis also revealed that age group, place of residence, educational status, religion, marital status, wealth index, sex of household head, reading newspaper, listening to radio, history of terminated pregnancy, current breastfeeding, and number of children were significantly associated with hormonal contraceptive utilization among reproductive-age women. This finding was in line with study that was conducted in Kenya [5], in Ethiopia [14], and in Uganda [18].

According to the study’s results, women between the ages of 15 and 19 were more likely than those between the ages of 35 and 39 to use modern contraceptives. This finding was in line with study that was conducted in Uganda [18], and in sub-Saharan countries [19]. It is possible that infrequent sexual activity and the conviction that there is no risk of pregnancy contribute to the lower likelihood of using modern contraception as people age [20, 21]. It is possible that women between the ages of 15 and 19 are still in school and are in the early stages of their reproductive lives. In order to meet their academic objectives, they might use contraception given their education and lack of desire to start a family.

Women of reproductive age who lived in urban areas were more likely than those who lived in rural areas to use modern contraceptives. This finding was in line with study that was conducted in Yemen [22], in Ethiopia [23–25] and in Pakistan [26]. This discrepancy may have existed due to the great availability of family planning services and the rising number of health institutions in urban areas. However, most women face several barriers to obtaining and using modern contraceptives, particularly those who live in rural areas [27] such as, low educational attainment, low economic status, deep rooted cultural belief [28], poor spousal communication, sociocultural norms, the husband’s role as the primary decision-maker, fear of side-effects, a lack of knowledge [29, 30], long distances to healthcare facilities, and inadequate stock of preferred types of modern contraceptives [31, 32]. Additionally, rural women require a larger number of children to assist them in their fieldwork, which negatively impacts their use of modern contraceptive methods [33, 34].

Compared to women who were Catholic, the likelihood of modern contraceptive use was lower among Muslim women. Compared to women who were Catholic, the likelihood of modern contraceptive use was lower among Muslim women. This finding was in line with studies that were conducted in Ghana [35], in Liberia [36]. This variations could be because religious resistance to their use may be more evident in Islam than in other religions [37, 38]. Additionally, most people who believed that family planning was incompatible with their beliefs declared that they had a duty to have as many children as God would allow them to have. In contrast to this, others thought family planning was appropriate given their moral obligation to raise and safeguard their children by reducing the number of children [38].

Married women were more likely than single women to use modern contraceptives. These findings were in line with studies that were conducted in Ethiopia [14], in Tanzania [39], and in Ethiopia [40]. The possible explanation is that women who were never in a union might not be in any sexual relationship, therefore resulting in their low use of modern contraceptives. Furthermore, from the perspective of males as a potential factor that influences the usage of modern contraceptives for fertility control, these differences may have arisen [41].

The likelihood of using modern contraceptives was higher among women with a middle wealth index than among those with a low wealth index. This finding was in line with the studies which were conducted in Nigeria [42], and in Ethiopia [43]. The contributing factors for these disparities were that the usage of contraceptives has a financial cost associated with it; women with a middle wealth index might be able to avoid any financial barriers to using modern contraceptives, while poor women might not [44, 45]; the level of household wealth has a significant impact on access to education, basic healthcare services, and health information [46].

In comparison, women who read magazines and listed radio stations were more likely to use modern contraceptives. This finding was in line with studies that were conducted in sub-Saharan Africa [47], in Ethiopia [48], Sierra Leone [49], Senegal [50] and Nigeria [51]. This may be because the media can enormously impact increasing awareness, intention, and use of reversible modern methods. After all, it constantly provides women with information and encouragement to continue using contraceptives.

Comparatively speaking, women who had previously terminated a pregnancy used modern contraception more frequently than those who had not. This finding was in line with a study that was conducted in Ethiopia [52]. This could be because women who have had their pregnancies terminated tend to use contraceptives less frequently. After all, they intend to become pregnant.

Compared to breastfeeding women, non-breastfeeding women were more likely to use modern contraceptives. This finding was in line with a study that was conducted in Ethiopia [53]. Menstruation not returning or low risk of fertility; fear of side effects; spouse disapproval; and fear of changing the composition of breast milk were the reasons for not using contraceptives [54–56].. Additionally, it is possible that this difference could be attributed to exclusive breastfeeding, which is a natural form of birth control. When a woman gives birth to her child, her body naturally stops ovulating if she exclusively breastfeeds and feeds her baby at least every four hours during the day and every six hours at night. If a woman misses her ovulation, she cannot become pregnant.

Women who had more than two children were more likely than those who had less than or equal to two children to use modern contraceptives. This finding was in line with studies that were conducted in Ethiopia [57], in Uganda, Malawi [58] and north west Ethiopia [59]. This may be the case because it may be necessary for women who are childless to have children to reach the optimal number of children. On the other hand, a possible reason might be that women who have achieved the desired number of children want to space out or limit further pregnancies by using modern contraceptives. One explanation for this could be that women who have already had two or more children are more likely to want to use modern contraceptives to prevent unplanned pregnancies because they are more aware of the difficulties and responsibilities that come with raising a child. Additionally, women without children might not feel as comfortable using contraceptives because they lack pregnancy and childbirth experience. Another argument is that women with two or more children might have better access to and knowledge of contemporary contraceptives through community outreach programs or healthcare providers, while women without children might not have as many opportunities to learn about and obtain these resources.

### Conclusions and recommendations

The factors that had a positive association with modern contraceptives were being married, living in an urban area, having more than two children, being in a female-headed household, having a middle-class income index, reading periodicals, listening to the radio, and not breastfeeding. However, using modern contraceptives has a negative association with being Muslim, between the ages of 35 and 39, and having a history of miscarriages. This suggests that addressing socioeconomic, geographic, and cultural hurdles may increase the efficiency of modern contraceptive use. As a result of this, the Kenyan government, along with other stakeholders, should greatly strengthen its efforts to disseminate information about the use of modern contraceptives and their importance for the health of mothers and their children. Special attention should be given to women who are in the lowest quintile of household income, live in rural areas, have a history of miscarriages, and have never attended formal education.

### Strengths and limitations of this study

- The DHS has a similar design with identical variables in a different environment; the result may, therefore, apply to other similar locations.
- The study used a sufficiently large sample size at the national level to ensure its representativeness.
- Recall bias is one of the potential drawbacks, especially for retrospective data based on experiences.
- The magnitude of the bias is often unknown, and correcting for the bias is difficult.
- Since this study was a cross-sectional study, it doesn’t show temporal relationships between independent and dependent variables.

## Data Availability

No datasets were generated or analysed during the current study.
